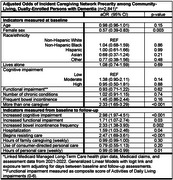# Predicting Caregiving Network Stability for Community‐Living Persons with Dementia

**DOI:** 10.1002/alz70858_097637

**Published:** 2025-12-24

**Authors:** Julia G Burgdorf, David Russell, Chanee D Fabius, Katherine Miller, Jennifer Reckrey

**Affiliations:** ^1^ Center for Home Care Policy & Research, New York, NY, USA; ^2^ Appalachian State University Department of Sociology, Boone, NC, USA; ^3^ Johns Hopkins Bloomberg School of Public Health, Baltimore, MD, USA; ^4^ New York University Langone Health, New York, NY, USA

## Abstract

**Background:**

Most Persons with Dementia (PwD) in the U.S. live in the community, relying on family/unpaid caregiving networks to meet their care needs. Caregiving network precarity refers to insecurity or instability in these networks, defined as caregiver(s) being unable or unwilling to continue in their role. While emerging evidence suggests that these networks are dynamic (i.e., members and roles change over time), little is currently known regarding factors associated with network precarity. This information is particularly relevant in the case of “dually‐enrolled” PwD: individuals who qualify for Medicaid insurance due to limited financial resources and who have greater social vulnerabilities and experience higher rates of adverse outcomes.

**Method:**

Using longitudinal 2021‐2022 linked health plan, Medicaid claims, and clinical assessment data for a diverse sample of community‐living, dually‐enrolled PwD (*n* = 2,841), we identify factors associated with caregiver reports of being unable or unwilling to continue providing care. We estimated adjusted odds of incident network precarity using multivariable Generalized Linear Models with a logit link, binomial family, and an exposure term to account for time under observation.

**Result:**

PwD in our sample had a mean age of 83.8 years (SD=8.4), 78.0% were female, 28.4% were Non‐Hispanic White, 32.4% were Hispanic, 18.7% were Non‐Hispanic Black, and 12.8% were Asian. In adjusted models, declines in health status were strongly associated with caregiver(s) reporting being unable/unwilling to continue providing care. PwD were more likely to experience incident caregiving network precarity if the PwD had recently experienced an increase in cognitive impairment (aOR: 2.98; 95% CI: 1.97‐4.51), functional impairment (aOR: 1.71; 95% CI: 1.07‐2.74), or bowel incontinence frequency (aOR: 2.33; 95% CI: 1.38‐3.93), or began resisting care (aOR: 2.47; 95% CI: 1.69‐3.61).

**Conclusion:**

Findings highlight the importance of identifying and addressing shifts in health and functional status for PwD and offering targeted supports to caregivers during these key inflection points. Better supporting dementia caregivers, particularly as they are faced with increasing or changing demands related to their caregiving role, is a critical component to facilitating stable and effective dementia care for community‐living PwD.